# BENIGN AND MALIGNANT SKIN LESIONS IN RENAL TRANSPLANT RECIPIENTS

**DOI:** 10.4103/0019-5154.55634

**Published:** 2009

**Authors:** H Ghaninejad, A H Ehsani, M Ghiasi, P Noormohammadpour, E Najafi, G Naderi, M Ganji, M Mirnezami, R Nezami, P Kiani

**Affiliations:** *From the Department of Dermatology, Razi Hospital, Tehran University of Medical Sciences, Tehran, Iran.*; 1 *From the Department of Nephrology, Shariati Hospital, Tehran University of Medical Sciences, Tehran, Iran.*

**Keywords:** *Basal cell carcinoma*, *benign*, *Kaposi sarcoma*, *malignant*, *renal transplantation*, *squamous cell carcinomas*, *skin lesions*

## Abstract

**Background:**

Skin lesions – benign and malignant – occur frequently in organ transplant recipients receiving long-term immunosuppressive therapy. These patients are at greater risk of skin cancers.

**Aims:**

To study dermatologic problems in renal transplant recipients (RTRs).

**Methods:**

One hundred patients (53 men and 47 women) were consecutively examined for benign and malignant skin complications since transplantation in Razi Hospital in Tehran Medical University. The main immunosuppressive therapy regimen in these patients was a combination of prednisolone, azathioprine, and cyclosporine.

**Results:**

The early and most common complication was cosmetic side effects that occurred in 98% patients. Skin infections occurred in 83% of the patients and most of them were viral infections (65%), especially of human papilloma viruses (HPVs) in 40% of the patients. We found six cases of malignancy in these patients in that four cases were skin cancers, including one case of SCC, one BCC, and two cases of Kaposi's sarcoma. Dermatologic problems occur most frequently in RTRs, especially skin cancers which have higher frequency in these patients than general population, particularly, Kaposi sarcoma. Sun exposure has an important role in developing epithelial skin cancers following transplantation. The age of developing skin cancer in these patients was early than normal population.

**Conclusion:**

Our results emphasize the importance of dermatologic examinations and monitoring RTRs to obtain an early diagnosis and treatment of cutaneous manifestations.

## Introduction

Organ transplantation is one of the miracles of modern medicine. Renal transplantation is the treatment of choice in endstage renal disease (ESRD).[1] Improvement in surgical techniques and advances in immunosuppressive therapy have led to a marked increase in the number of organ transplants worldwide and to long-term survival after transplantation. Immunosuppressive drugs are of great importance in adequate graft function; however, they produce important inhibitory effects on immune defense mechanisms favoring the occurrence of complications, particularly infections and malignancies. Varied cutaneous viral, fungal, and bacterial infections as well as an increased frequency of skin cancers have been observed among organ transplant recipients.[2–5] With the advent of new immunosuppressive therapies and different treatment regimens, there is an increasing need for a multidisciplinary approach to blanching the risks and benefits of these medications to the individual transplant recipient.[6]

The incidence of malignancy increases after renal transplantation. Skin cancer is the most frequent, and is seen in 3-10% of RTRs.[7–9] These cancers include squamous cell carcinomas (SCC), basal cell carcinoma (BCC), Bowen's disease Kaposi sarcoma, and probably malignant melanoma.[10] In organ transplant recipients skin cancers are usually multiple and invasive and have greater risk of recurrence after treatment. The risk of metastasis is also high in skin cancers of these patients.[9,11,12]

We examined the incidence and clinical spectrum of cutaneous manifestations occurring in RTRs.

## Materials and Methods

This descriptive study was done on RTRs followed up by three nephrologists after transplantation (from December 2000 to May 2002). One hundred patients underwent regular clinical follow-up visit every three months by nephrologists and full skin examination every six months by dermatologist at Razi Hospital in Tehran University. Beyond scheduled visits, patients were also referred for dermatological consultation when a suspicious lesion or eruption was noted by the physician or the patient. In addition to dermatological examination, a standardized questionnaire was administered to all patients to obtain a detailed personal history, including information on age, occupation, cause of renal failure, date of renal transplant, and current immunosuppressive drug regimen.

Complete dermatologic history was obtained and patients with a suspicious history of malignant or premalignant skin lesions before transplantation were excluded from the study.

In addition to these regular visits, information of past medical history of all patients that was recorded in their medical documents about cutaneous lesions after transplantation was also considered in this study.

## Results

A total of 100 RTRs (53 males and 47 females), aged 14-67 years (mean: 37.8 years), were enrolled in this study. Patients had undergone renal transplantation between 3-140 months (mean: 52 months) before the first dermatological observation. Time after transplant is presented in Table 1. Occupation was of indoor type in 85 patients and outdoor in 15 patients. Four patients had skin type-2 (very sensitive, always burn), 39 patients had skin type-3 (sensitive, burn minimally), and 57 patients had skin type-4 (moderately sensitive, burn minimally). Immunosuppressive regimen in 80 patients was prednisolone, cyclosporine, and azathioprine (P+C+A) and in 20 patients it was prednisolone and cyclosporine (P+C). Ninety eight patients developed one or more cutaneous complication post-transplantation. Cutaneous complications in these patients were divided to three groups that are detailed as follows.

**Table 1:** Number of patients and post-transplantation time

Number of patients	Time after transplantation (years)
14	<1
46	1-4
34	4-8
6	8-12

### Cosmetic complications

This group was the most common cutaneous complication post-transplantation and was seen in 98 patients [Table 2]. Among them hypertrichosis was the most common that affected trunk and upper limb with greater severity.

**Table 2:** Cosmetic complication post renal transplantation

Complication	Percent
Hypertrichosis	91
Cushingoid face	70
Acne	60
Gingival hyperplasia	44
Ecchymosis	28
Alopecia	10
Hyperhidrosis	9
Hirsutism	9
Stria	7
Telangiectasia	6
Sebaceous hyperplasia	2

### Skin infections

Eighty three patients demonstrated at least one episode of viral, bacterial, or fungal skin infection [Table 3]. The most common skin infection in these patients was viral and among them warts were most frequent (40%). In eight patients warts were detected in the first year of transplantation, in 10 cases between 1-3 years, and in 22 cases after third year of transplantation. Thirty three patients were receiving three-drug regimen and 7 patients two-drug regimen. HSV infection was seen in 32 patients and the majority of these patients was affected in the first month of transplantation. Fifty six patients were affected with one type of superficial fungal infections. Tinea versicolor (35%), frequently was detected between first and third year of transplantation, candidiasis (34%) was more frequent in the first month after transplantation.

**Table 3:** Type of skin infection post-transplantation

Infection	Percent
Wart	40
*Tinea versicolor*	35
*Herpes simplex*	34
Facial	32
Genital	2
Candidiasis	34
Oral	26
Genital	8
*Herpes zoster*	16
Dermatophytes	4
Infection	2
Furuncle	1
Erythrasma	1

### Malignant and premalignant lesions

Of six cases of malignancy diagnosed in these patients, four cases were of skin cancer [Table 4]. The two remaining cases were transitional cell carcinoma of bladder in one case and non-Hodgkin lymphoma in the other. There was also, one case of actinic keratoses (premalignant) and three cases of seborrheic keratoses. BCC was detected in a 39-year-old man, 48 months post-transplantation. Skin type of this patient was 4 and the patient was receiving three-drug immunosuppressive regimen (P+C+A). The site of BCC was on the nose. SCC was diagnosed in a 42-year-old male 48 months after transplantation. His skin type was 3 and he was on two-drug immunosuppressive regimen (P+C). The site of SCC was on the cheek. Two cases of Kaposi's sarcoma were found in two male patients aged 51 and 58 years. Their skin types were 3 and 4, respectively. The lesion in the first patient was diagnosed 24 months and in the second patient one month post-transplantation. The immunosuppressive regimens of both patients were two drugs (P+C).

**Table 4:** Premalignant and malignant skin lesions

Lesions	Percent
Actinic keratosis	1
Squamous cell carcinoma	1
Basal cell carcinoma	1
Kaposi's sarcoma	2

## Discussion

In this study, we considered relative frequency of different skin complications in our country RTRs. The most frequent skin lesions in these patients were any of the above discussed cosmetic complications affecting 98% patients. The majority of these problems were known side effects of steroid, but cyclosporine has also potential of inducing different cosmetic problems, including hypertrichosis, gingival overgrowth, sebaceous hyperplasia, and hair- like follicular keratoses.[13–15] In recent years, some new cosmetic side effects of cyclosporine have been reported that had not been seen in the past such as cyclosporine-induced folliculodystrophy.[16] In the early period of post-transplantation high doses of these drugs resulted in increased prevalence of cosmetic complications.[17]

The majority of patients was affected with one or more skin infections. The most common skin infections in these patients were viral infections. Warts were the most common viral infection in our series as well as in previously reported studies.[2,17,18] Warts and tinea versicolor were more common after the first year of transplantation rather than during the first year, but HSV infections and candidiasis were more common in the early period after transplantation. Overall, regarding the time of infections we could state that zoster, herpes simplex, and candidiasis are early onset infections and warts and versicolor are late onset infections. These findings are similar to the results of Sandhue *et al.,* and Formicone *et al.,* studies.[2,18]

Malignancies are a well-recognized complication of renal transplantation. Although this problem is well studied in developed countries, less is known about it in developing countries and because of geographic and ethnic variations, further investigations about this problem is a necessity.[19] Skin cancers are the most common malignant conditions in organ transplant recipients and the risk of them is related to the degree of immunosuppression caused by long-term immunosuppressive therapy.[18,20] In patients receiving cyclosporine the risk of skin cancer is 6–8% in comparison with 2.2-5.5% in patients receiving other immunosuppressive drugs.[10] Similar to the results of previous studies in our study skin cancers were the most common malignancy in RTRs.[11,21] In the majority of previous studies, SCC and BCC account for more than 90% of all skin cancers,[5,18] but in our study Kaposi sarcoma was the most common form of skin cancer, detected in two patients. However, similar to our results Kaposi's sarcoma described as most common post-transplant cancer in developing countries are two previous studies.[19,22] In normal population BCC to SCC ratio is 4:1 but in organ transplant recipients the incidence of SCC increases and this ratio reverses. A linear increase in incidence has been reported for BCC, while for SCC the increased incidence was initially slower but later exponential.[18] In our study, the ratio of SCC to BCC was 1:1, but comparing with normal population SCC was more frequent. The equal incidence of BCC and SCC observed in our patients might be due to the relatively short period after transplantation. However, a high BCC/SCC ratio has been previously described in Mediterranean populations of RTRs.[23] Additionally, in our study both patients developed skin cancers 48 months after transplantation and that this interval was shorter comparing previous studies. In Ramsay *et al.,* study the mean duration between transplantation and development of malignancy was five years.[24] The genetic background of Mediterranean patients, sun exposure habits, and phenotypic characteristics could explain the higher incidence of BCC and earlier development of skin cancers in these populations. Both SCC and BCC patients in our study had excessive sun exposure because of their occupations (in outdoor). This indicates that UV radiation may have had an important role in developing epithelial skin cancers in these patients (both lesions situated on the face). One of most important factor for decreasing the risk of skin cancers in organ transplant recipients is sun avoidance. In one study on 151 RTRs only 40.4% of patients knew that the development of skin cancer is connected with the exposure to sunlight and more than a half of the patients did not apply any sun protection.[25]

On the basis of our results and previous studies, clinicians should be aware of the importance of careful dermatological monitoring of RTRs for early diagnosis and treatment of cutaneous diseases. In addition, there is necessity of education concerning the harmfulness of sunlight and the methods of sun protection among RTRs.
